# Ceramide metabolism associated with chronic dietary nutrient surplus and diminished insulin sensitivity in the liver, muscle, and adipose tissue of cattle

**DOI:** 10.3389/fphys.2022.958837

**Published:** 2022-08-08

**Authors:** Ákos Kenéz, Sonja Christiane Bäßler, Ezequiel Jorge-Smeding, Korinna Huber

**Affiliations:** ^1^ Department of Infectious Diseases and Public Health, Jockey Club College of Veterinary Medicine and Life Sciences, City University of Hong Kong, Kowloon, Hong Kong SAR, China; ^2^ Institute of Animal Science, Faculty of Agricultural Sciences, University of Hohenheim, Stuttgart, Germany

**Keywords:** sphingolipids, ceramide, insulin resistance, tissue metabolomics, obesity, metabolic inflammation

## Abstract

High dietary energy and protein supply is common practice in livestock nutrition, aiming to maximize growth and production performance. However, a chronic nutritional surplus induces obesity, promotes insulin insensitivity, and triggers low-grade inflammation. Thirty Holstein bulls were randomly assigned to two groups, low energy and protein (LEP), and high energy and protein (HEP) intake, provided from the 13th to the 20th month of life. Body weight, carcass composition, laminitis score, and circulating insulin and glucose concentrations were assessed. The expression and extent of phosphorylation of insulin signaling proteins were measured in the liver, muscle, and adipose tissue. The sphingolipid metabolome was quantified by a targeted liquid chromatography-mass spectrometry based metabolomics approach. The HEP bulls were obese, had hyperinsulinemia with euglycemia, and expressed clinical signs of chronic laminitis. In the liver, protein kinase B (PKB) phosphorylation was decreased and this was associated with a higher tissue concentration of ceramide 16:0, a sphingolipid that diminishes insulin action by dephosphorylating PKB. In the adipose tissue, insulin receptor expression was lower in HEP bulls, associated with higher concentration of hexosylceramide, which reduces the abundance of functional insulin receptors. Our findings confirm that diet-induced metabolic inflammation triggers ceramide accumulation and disturbs insulin signaling. As insulin insensitivity exacerbates metabolic inflammation, this self-reinforcing cycle could explain the deterioration of metabolic health apparent as chronic laminitis. By demonstrating molecular relationships between insulin signaling and sphingolipid metabolism in three major tissues, our data extend our mechanistic understanding of the role of ceramides in diet-induced metabolic inflammation.

## Introduction

There is a well-documented causal relationship between chronic consumption of hypercaloric diets and insulin resistance demonstrated in humans and various animal models (Deer et al., 2015; Sah et al., 2016). Continuous or recurrent nutrient surplus induces dyslipidemia, triglyceride deposition, and adipocyte hypertrophy, leading to obesity (Gregor and Hotamisligil, 2011). However, due to the complexity of metabolic events initiated, including the highly integrated cellular responses to nutrient excess in various tissues and organ systems, mapping the multifaceted contributing factors of metabolic dysfunction remains challenging (Wisse et al., 2007). There is a chronic low-grade inflammation developing in obesity, mediated by inflammatory cytokines such as tumor necrosis factor-alpha (TNF-α), interleukin 6 (IL-6), monocyte chemoattractant protein-1 (MCP-1), and interleukin 8 (IL-8) (Zeyda and Stulnig, 2009). This has been termed metabolic inflammation or “metaflammation,” a common endpoint of converging signaling pathways activated by chronic nutrient excess (Gregor and Hotamisligil, 2011; Hotamisligil, 2017). Multiple interconnected molecular mechanisms have been identified underlying the interference of these inflammatory signals with insulin signaling, including the induction of ceramide synthesis, eventually resulting in insulin resistance (Dali-Youcef et al., 2013). Insulin resistance is determined by impaired sensitivity to insulin in its main target organs, i.e., adipose tissue, liver, and muscle (Zeyda and Stulnig, 2009). Nevertheless, the consequences of such insulin dysregulation also include further metabolic deterioration, triggering an unresolved vicious cycle (Hotamisligil, 2006).

Sphingolipid metabolites, including ceramide, interact with pro-inflammatory pathways and with insulin signaling. Inflammatory cytokines such as TNF-α released from hypertrophic adipocytes and M1 macrophages infiltrating adipose tissues in obesity induce *de novo* ceramide synthesis from palmitate and transformation of sphingomyelin into ceramide by sphingomyelinase activity (Holland and Summers, 2008). Similarly, ceramide accumulation is also induced in response to TLR-4 activation, Fas ligand, and oxidative stress in a variety of cell types (Sokolowska and Blachnio-Zabielska, 2019). Ceramides, particularly long-chain ceramides such as C16:0 and C18:0 antagonize insulin signaling by diminishing Akt (PKB) phosphorylation, a key step for GLUT4 translocation (Meikle and Summers, 2017). In addition, hexosylceramide derivatives, particularly gangliosides, antagonize insulin signaling by displacing the insulin receptor and inhibiting receptor tyrosine phosphorylation (Holland and Summers, 2008). Thereby, ceramides counteract cellular glucose uptake and interfere with nutrient storage, in addition to promoting proinflammatory cytokine production, disrupting hepatic lipid metabolism, and enhancing cell death (Sokolowska and Blachnio-Zabielska, 2019). In addition, the insulin-sensitizing effect of adiponectin was attributed to its ceramidase activity, further highlighting the central role of ceramides in regulating insulin sensitivity (Reibe-Pal and Febbraio, 2017).

From an evolutionary point of view, a close cross-talk between immune function and metabolic response is a highly desired trait (Hotamisligil, 2006). In general, inflammation is a physiological mechanism to help restore metabolic homeostasis and functional integrity of organs and tissues (Hotamisligil, 2017). Furthermore, the organism needs to coordinate and redistribute nutrients during an inflammatory response, which is why the integration of pathogen- and nutrient-sensing pathways and the control of anabolic pathways are a meaningful biological strategy for survival under challenged conditions (Beutler, 2004). However, in contrast to temporarily diverting energy sources away from synthetic pathways during a pathogen-induced “classical” inflammation, the activation of these evolutionarily conserved systems during “metaflammation” contributes to the unresolved vicious cycle of metabolic deterioration induced by chronic nutrient surplus (Hotamisligil, 2006). Since obesity and the cluster of obesity-related metabolic disorders have reached epidemic proportions in humans, the molecular mechanism of metabolic dysregulation, including sphingolipid signaling during obesity-induced metaflammation have been extensively studied in humans and rodent models (Holland and Summers, 2008; Gregor and Hotamisligil, 2011; Meikle and Summers, 2017; Sokolowska and Blachnio-Zabielska, 2019; Summers et al., 2019). Metaflammation triggered by excessive lipid mobilization after over-conditioning, among other factors, has also been identified as a key component of metabolic disorders commonly occurring in modern-day high-performance dairy cows during the transition from gestation to lactation (Bradford et al., 2015). Presumably owing to the evolutionary conserved regulation, underlying pathophysiological mechanisms of metabolic dysfunction (i.e., inflammatory signaling and inhibition of insulin signaling with an increase of ceramide mediators) largely overlap in these conditions between humans and cattle (Bradford et al., 2015; Rico et al., 2018; McFadden and Rico, 2019; Bradford and Swartz, 2020; McFadden, 2020).

We postulate that studying the relationship between chronic nutrient surplus, ceramide metabolism, and insulin signaling in cattle will extend our global understanding of cellular mechanisms driving metabolic health. We hypothesized that an intensive fattening regimen in bulls based on a high energy and protein diet would reduce insulin sensitivity by inducing a pro-inflammatory shift in the sphingolipid metabolome. Our objectives were to measure the abundance and phosphorylation level of key proteins of insulin signaling and nutrient sensing, as well as to quantify the concentration of various sphingolipids, including ceramides, in a metabolomics approach. By extending these measurements to the liver, skeletal muscle, and adipose tissue in Holstein fattening bulls, we aimed to map the effect of chronic nutrient surplus on the interplay between organs as well.

## Materials and methods

### Experimental design

The animal experiment was conducted at the Educational and Research Centre for Animal Husbandry, Hofgut Neumuehle (Muenchweiler a.d. Alsenz, Germany) as reported previously (Bäßler et al., 2021). All experimental procedures were approved by the Animal Ethics Committee of the Department for Animal Welfare Affairs (Landesuntersuchungsamt Rheinland-Pfalz, Koblenz, Germany) in agreement with the German Animal Welfare Act (permit number: G-17-20-070). Briefly, Holstein bulls were randomly assigned to a high energy and protein (HEP; *n* = 15) or a low energy and protein (LEP; *n* = 15) fattening regimen at an average age of 13 months and average body weight of 500 kg (HEP 506 ± 35 kg, LEP 499 ± 35 kg; mean ± SD). The experimental feeding lasted from 13 months of age until the time of slaughtering 7 months later. The sample size was consistent with commonly reported study designs in livestock metabolomics (Goldansaz et al., 2017).

### Experimental diet

The diets were formulated according to the high- (HEP) and low-end (LEP) of the range of recommendations of the Bavarian State Research Center for Agriculture (LfL) for Simmental fattening bulls. While the LEP diet consisted of silage only, the HEP diet consisted of silage and concentrate (a blend of ground corn, rapeseed meal, ground wheat, palm kernel meal, wheat bran, molasses, and soybean meal), accounting for an increased total sugar, total starch, and crude protein, as well as a decreased fiber content of the HEP diet, relative to LEP. The diets were fed as total mixed ration (TMR) and the nutrient composition of the TMR was determined as reported previously (Bäßler et al., 2021). The ingredients and chemical composition of the TMR were published previously (Bäßler et al., 2021) and included here in the Supplementary Table S1.

### Sample and data collection

At the end of the experimental feeding period, all bulls were slaughtered at an average age of 20 months, and live weight, carcass conformation, and fat class, and laminitis score were recorded upon slaughtering, and serum insulin and plasma glucose were measured as published previously (Bäßler et al., 2021). In addition, tissue samples of the liver (the ventral third of the diaphragmatic surface), muscle (musculus longissimus dorsi), and retroperitoneal adipose tissue (perirenal region, between the peritoneum and the abdominal muscles) and subcutaneous adipose tissue (at the tailhead region) were collected immediately after slaughtering. Tissue samples were trimmed of any connective tissue, cut into approximately 0.5 cm × 0.5 cm × 0.5 cm pieces, rinsed in ice-cold physiological saline solution, snap-frozen in liquid nitrogen, and stored at −80°C until analyses.

### Western blot analysis

The expression and phosphorylation of key proteins of the insulin signaling pathway were measured in liver, muscle, and retroperitoneal adipose tissue samples by Western blotting following our previously published protocol (Kenéz et al., 2019). The conditions of antibody detection for insulin receptor β (InsR), mechanistic target of rapamycin (mTOR), phosphorylated mTOR, protein kinase B (PKB), phosphorylated PKB, 5’ adenosine monophosphate-activated protein kinase α (AMPK), and phosphorylated AMPK are listed in Table 1.

**TABLE 1:** Primary and secondary antibodies used for western blot analyses.

Target antigen	Antibody	Dilution	Manufacturer	Buffer	Blocking agent
Insulin receptor β (InsR)	Rabbit anti InsR-β (4B8)	1:2,000	Cell Signaling Technology Inc. (Danvers, MA, United States)	5% bovine serum albumin	5% fat-free milk powder
Mechanistic target of rapamycin (mTOR)	Rabbit anti mTOR (7C10)	1:1,000	Cell Signaling Technology Inc.	5% bovine serum albumin	5% fat-free milk powder
Phosphorylated mechanistic target of rapamycin (Ser2448; p-mTOR)	Rabbit anti p-mTOR (Ser2448)	1:500	Cell Signaling Technology Inc.	5% bovine serum albumin	5% bovine serum albumin
Protein kinase b (PKB)	Rabbit anti AKT	1:2,000	Cell Signaling Technology Inc.	5% bovine serum albumin	5% fat-free milk powder
Phosphorylated protein kinase b (Ser473; p-PKB)	Rabbit anti p-AKT (Ser473) (D9E) XP	1:2,000	Cell Signaling Technology Inc.	5% bovine serum albumin	5% fat-free milk powder
5′ adenosine monophosphate-activated protein kinase α (AMPK- α)	Rabbit anti AMPK α	1:4,000	Bethyl Laboratories Inc. (Montgomery, TX, United States)	5% fat-free milk powder	5% fat-free milk powder
Phosphorylated 5′ adenosine monophosphate-activated protein kinase α (Thr172) (p-AMPK- α)	Rabbit anti p-AMPKα (Thr172) (40H9)	1:1,000	Cell Signaling Technology Inc.	5% fat-free milk powder	5% fat-free milk powder
Rabbit IgG (secondary antibody)	Goat anti-rabbit IgG, HRP-linked	1:2,000	Cell Signaling Technology Inc.	5% fat-free milk powder	5% fat-free milk powder

### Metabolomics analysis

The sphingolipid metabolome was quantified by a targeted liquid chromatography-mass spectrometry (LC-MS) based metabolomics assay in the liver, muscle, and subcutaneous adipose tissue samples. This analysis was carried out at the UVic Node of The Metabolomics Innovation Centre (TMIC; Genome BC Proteomics Centre, The University of Victoria, Victoria, BC, Canada) following the previously published workflow (Leung et al., 2020). The 77 compounds targeted herein belonged to the following sphingolipid classes: 3-keto-sphinganine (3-KSpha), sphinganines (Spha), sphinganine-1-phosphates (Spha-P), dihydroceramides (dhCer), dihydroceramide-1-phosphates (dhCer-P), ceramides (Cer), ceramide-1-phosphates (Cer-P), galactosyl-ceramides (GalCer), glucosyl-ceramides (GlcCer), lactosyl-ceramides (LacCer), sphingomyelins (SM), dihydrosphingomyelins (dhSM), and sphingosines (Spho). In brief, 200 mg of the tissue samples were homogenized in 200 µl of water and subsequently mixed with methanol-chloroform (5:2, v/v, 19 µl/mg raw tissue) containing butylated hydroxytoluene (0.1 mg/ml). The sample was then sonicated (5 min) in an ice bath, and centrifuged (30 min, 4,000 × *g*, 10*°*C). The clear supernatant was collected and the precipitated pellet was extracted with chloroform (1:1, v/v, 10 µl/mg raw tissue). The clear supernatant was collected again, dried off under a nitrogen gas flow (30 C), and the residue was dissolved in methanol-chloroform (1:1, v/v, 30 µl/mg). The sphingolipid measurement was performed by ultra-performance liquid chromatography-tandem mass spectrometry in multiple reaction monitoring mode (UPLC-MS) using the positive (ESI+) and negative (ESI-) ion modes for sphingolipids and phosphorylated sphingolipids, respectively. Concentrations were calculated by peak area calibration curves with standard dilutions. Sphingolipid metabolites were filtered according to the limit of detection (LOD) of each feature, and only those features were retained that were effectively quantified (concentration > LOD) in at least 80% of the samples of either one of the experimental groups. Missing values were replaced by LOD/2 according to our previously published workflow (Leung et al., 2021).

### Statistical analysis

Live weight, carcass class, laminitis score, circulating insulin and glucose, and protein abundance and phosphorylation data were compared between the HEP and LEP treatments by unpaired Student’s *t*-test in GraphPad Prism (version 9.3). Sphingolipid metabolome data were analyzed by partial least-squares discriminant analysis (PLS-DA). The PLS-DA models were evaluated by their goodness of fit (R^2^) and prediction quality index (Q^2^). R^2^ and Q^2^ values of 1.0 represent the best possible models and the models were considered valid herein if Q^2^ > 0 for the first component on the first run. Within each PLS-DA model, the metabolites’ contribution to the model was assessed by their variable importance in projection (VIP) scores (greater values indicating a more significant contribution). Metabolome data were also subjected to an unpaired Student’s *t*-test with false discovery rate (FDR) correction, and heatmap combined with hierarchical cluster analysis in MetaboAnalyst (version 5.0) (Pang et al., 2021). Further, the associations between insulin signaling protein expression and sphingolipid concentrations were evaluated by principal component analysis (PCA) loading plots according to (Jorge-Smeding et al., 2021).

## Results

### Body condition, laminitis scores, and circulating insulin and glucose concentrations

**FIGURE 1:**
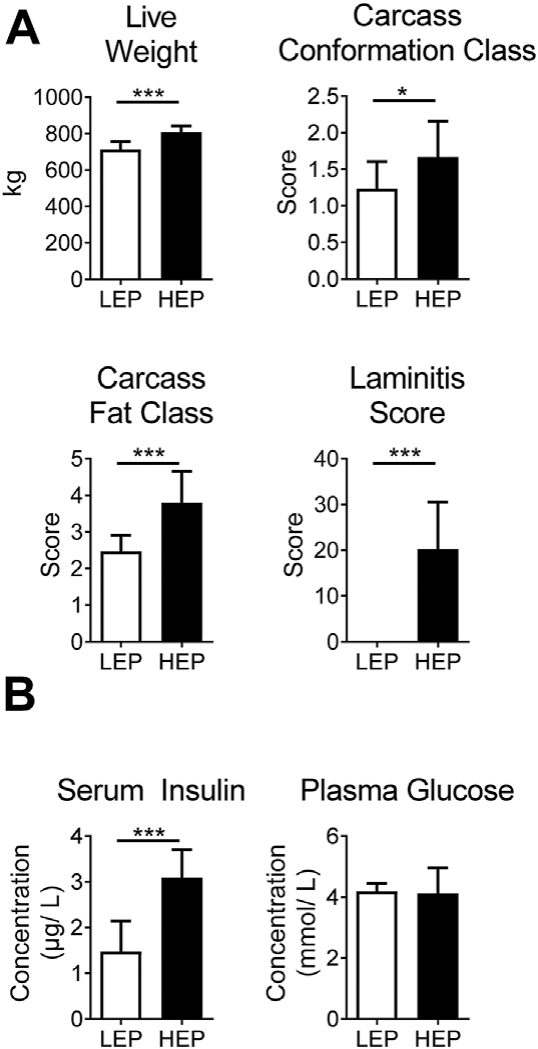
**(A)** Body weight, carcass classes and laminitis score in HEP and LEP fed bulls. **(B)** Serum insulin and plasma glucose of HEP and LEP fed bulls, as published previously (Bäßler et al., 2021). Means ± SD; *n* = 15.

Bulls in the HEP group had significantly higher final body weight (*p* < 0.001) upon slaughtering, as well as higher carcass conformation (*p* = 0.01) and fat class (*p* < 0.001) than the LEP bulls (Figure 1A), as published previously (Bäßler et al., 2021). Furthermore, HEP bulls had a high laminitis score, while LEP bulls scored zero on the same scale, resulting in a greater average score for the HEP bulls (*p* < 0.001). Bulls of the HEP group had basal hyperinsulinemia with euglycemia, in contrast to LEP (serum insulin *p* < 0.001; plasma glucose *p* = 0.79) (Figure 1B).

### Insulin signaling

**FIGURE 2:**
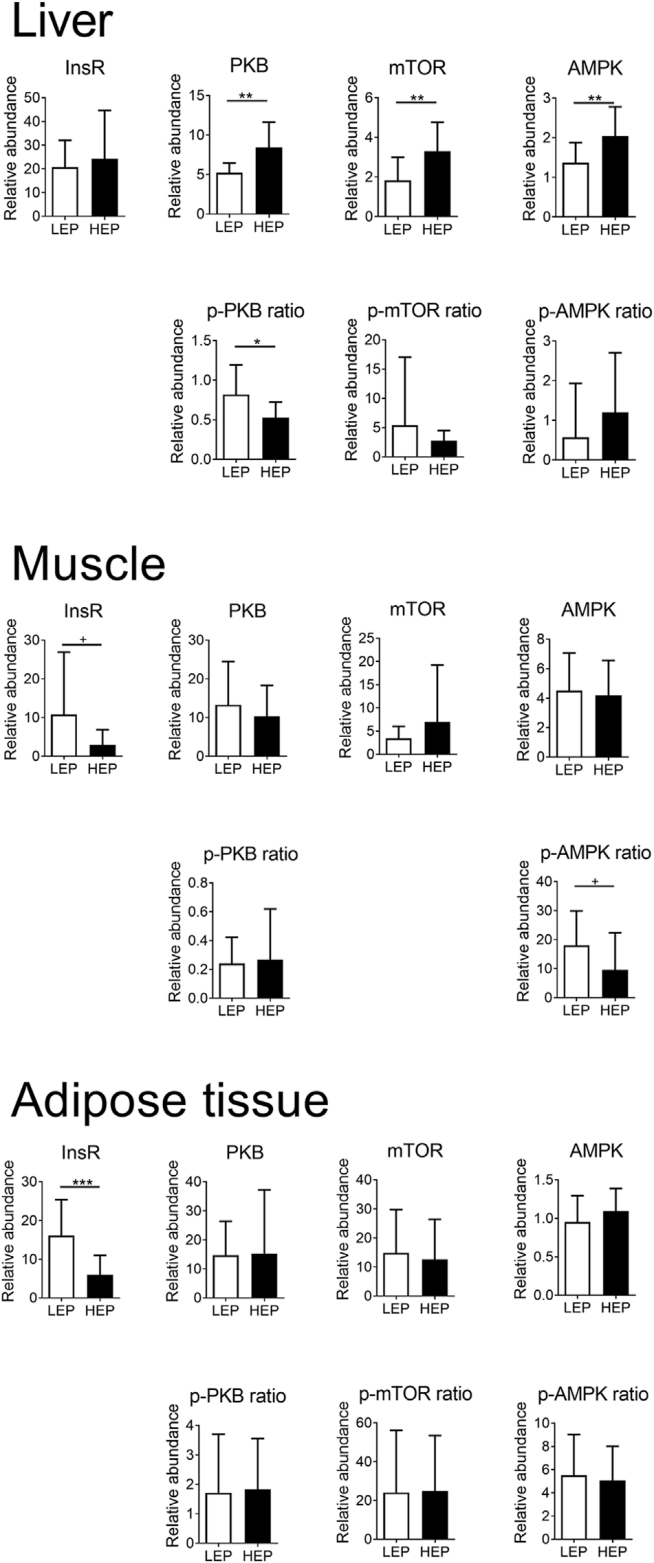
Protein expression and phosphorylation of key components of the insulin signaling pathway in liver, muscle, and retroperitoneal adipose tissue. Insulin receptor β (InsR), mechanistic target of rapamycin (mTOR), protein kinase B (PKB), 5′ adenosine monophosphate-activated protein kinase α (AMPK), and their phosphorylated forms were detected by Western blotting. Means ± SD; *n* = 15.

Figure 2 shows the extent of expression and phosphorylation of the insulin signaling proteins in all tissues, comparing HEP and LEP. The expression of InsR was decreased in the retroperitoneal adipose tissue (*p* < 0.001) and tended to be decreased (*p* = 0.07) in the muscle in the HEP group, compared with LEP. The downstream signaling elements of the insulin pathway were similar between HEP and LEP in the muscle and the retroperitoneal adipose tissue (all *p* > 0.1), however, a tendency for lower AMPK phosphorylation ratio was detected in HEP, compared with LEP (*p* = 0.07). The abundance of InsR protein was not different between HEP and LEP in the liver, however, greater abundances of PKB, mTOR, and AMPK protein (*p* = 0.001, *p* = 0.005, and *p* = 0.007, respectively) were detected, concurrently with lower phosphorylation ratio of PKB (*p* = 0.01) in HEP, compared with LEP. Representative Western blot images are shown in Supplementary Figure S1.

### Sphingolipid metabolome

**FIGURE 3:**
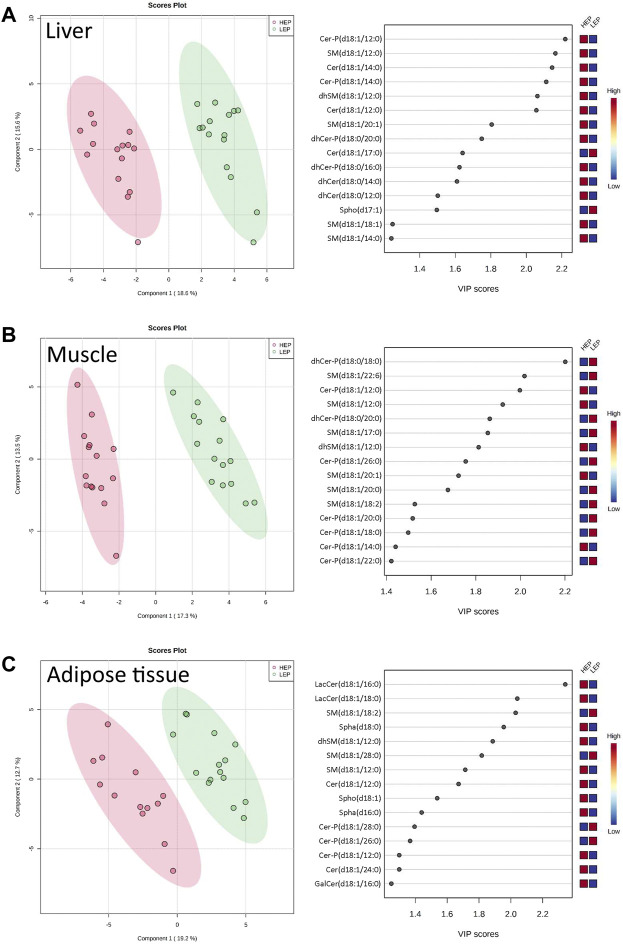
Partial least squares-discriminant analysis (PLS-DA) scores plots with variable importance in projection (VIP) scores of sphingolipid metabolome profiles of **(A)** liver, **(B)** muscle, and **(C)** subcutaneous adipose tissue of bulls on HEP and LEP dietary regimens (*n* = 15). Spha, sphinganines; dhCer, dihydroceramides; dhCer-P, dihydroceramide-1-phosphates; Cer, ceramides; Cer-P, ceramide-1-phosphates; GalCer, galactosyl-ceramides; LacCer, lactosyl-ceramides; SM, sphingomyelins; dhSM, dihydrosphingomyelins; Spho: sphingosines.

A total of 71 out of the targeted 77 sphingolipid species were effectively quantified (concentrations higher than the lower limit of detection in at least 80% of the samples). These were assigned to three main sphingolipid pathways: *de novo* synthesis (3-KSph, Spha, Spha-P, dhCer, dhCer-P, Cer), sphingomyelinase pathway (SM, dhSM), and salvage pathway (GalCer, GlcCer, LacCer, Cer-P, Spho) (Supplementary Table S2). The sphingolipid metabolome profiles were significantly separated between HEP and LEP in all three tissues, as assessed by PLS-DA (Figure 3), which had valid models with high values for fitness and prediction quality for the three tissues (liver: *R* ^*2*^ = 0.82, *Q* ^*2*^ = 0.74; muscle: *R* ^*2*^ = 0.76, *Q* ^*2*^ = 0.68; adipose tissue: *R* ^*2*^ = 0.93, *Q* ^*2*^ = 0.82). The heatmaps in Supplementary Figure S2 show the relative concentration of individual sphingolipids that were significantly different between HEP and LEP (*t*-test, FDR corrected *p* < 0.05). A total of 20, 21, and 11 sphingolipid species had different tissue concentrations in the liver, muscle, and adipose tissue, respectively (Supplementary Figure S2). When categorizing and summing the individual sphingolipid species according to their chemical characteristics, several differences were detected between LEP and HEP bulls in the three tissues (Table 2). Specifically, Cer C16:0 (*p* = 0.009) and total dhCer-P (*p* < 0.001) had greater concentrations in HEP than LEP in the liver. In the muscle, a greater ratio of Cer:SM (*p* = 0.007) and lower concentrations of total dhCer (*p* = 0.037), total dhCer-P (*p* = 0.001), and total SM (*p* = 0.001) were found in HEP than LEP. In the subcutaneous adipose tissue, greater concentrations of Cer 24:0 (*p* = 0.006), total hexosylceramide (*p* = 0.003), total Spha (*p* = 0.001), and Spho (*p* = 0.005) were observed in HEP, compared with LEP.

**TABLE 2:** Insulin signaling protein expression and phosphorylation and abundance of ceramides C16:0, C18:0, and C24:0 and summative sphingolipid classes in the liver, skeletal muscle, and subcutaneous adipose tissue (sphingolipids) or retroperitoneal adipose tissue (insulin signaling proteins) of bulls fed either the low energy and protein (LEP) or the high energy and protein (HEP) diet. All concentrations all expressed as nmol/g tissue.

Feature	Liver					Muscle					Adipose tissue				
	LEP	±SD	HEP	±SD	*p*-value	LEP	±SD	HEP	±SD	*p*-value	LEP	±SD	HEP	±SD	*p*-value
Cer 16:0	1.02	0.08	1.17	0.18	0.009	0.016	0.005	0.014	0.005	0.344	0.462	0.120	0.562	0.305	0.251
Cer 18:0	0.028	0.008	0.025	0.005	0.056	0.143	0.022	0.136	0.027	0.476	0.044	0.014	0.078	0.094	0.171
Cer 24:0	0.369	0.161	0.309	0.116	0.251	0.174	0.059	0.208	0.077	0.184	0.181	0.035	0.260	0.095	0.006
Total Cer	2.21	0.32	2.29	0.35	0.530	0.211	0.049	0.239	0.060	0.202	0.985	0.189	1.226	0.492	0.092
Total Cer-P	78.4	6.2	82.6	6.6	0.083	56.0	4.2	53.5	2.7	0.065	63.3	9.7	64.7	8.8	0.696
Total dhCer	1.27	0.36	1.28	0.27	0.959	0.057	0.040	0.032	0.015	0.037	0.203	0.150	0.252	0.135	0.376
Total dhCer-P	4.30	0.62	5.33	0.78	< 0.001	23.6	6.0	17.4	2.6	0.001	7.98	2.39	8.80	1.77	0.319
Total HexCer	0.75	0.20	0.65	0.17	0.403	0.21	0.06	0.26	0.08	0.087	0.41	0.13	0.63	0.22	0.003
Total SM	129.8	19.8	126.1	13.4	0.554	67.7	5.5	59.2	7.5	0.001	86.8	9.6	91.9	16.1	0.309
Total sphinganines	0.206	0.222	0.106	0.080	0.108	0.066	0.018	0.058	0.011	0.186	0.057	0.016	0.095	0.034	0.001
Total sphingosines	0.960	0.928	0.486	0.299	0.070	0.431	0.103	0.452	0.083	0.537	0.167	0.061	0.282	0.131	0.005
Total Cer:SM	0.017	0.002	0.018	0.002	0.153	0.0031	0.0008	0.0040	0.0008	0.007	0.011	0.003	0.014	0.007	0.238

Cer, ceramides; Cer-P, ceramide-phosphates; dhCer, dihydroceramides; dhCer-P, dihydroceramide-1-phosphates; HexCer, hexosylceramides (sum of glucosyl-, galactosyl-, and lactosylceramides); SM, sphingomyelins.

**FIGURE 4:**
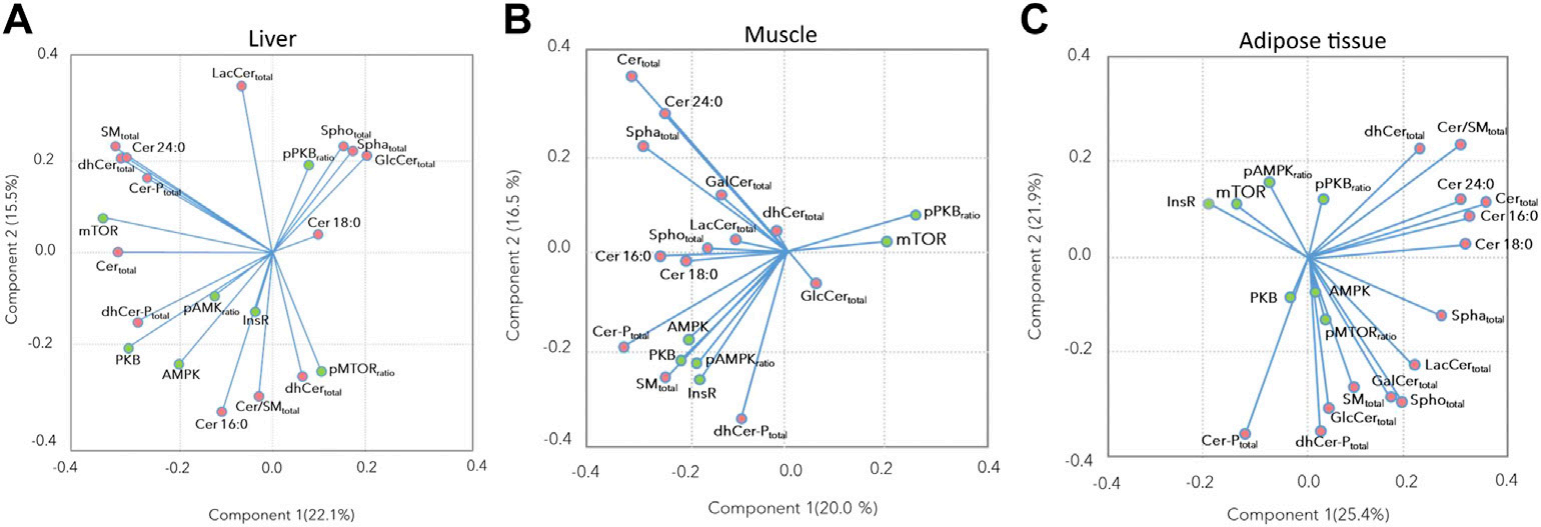
Principal component analysis loading plots for **(A)** liver, **(B)** muscle, and **(C)** subcutaneous and retroperitoneal adipose tissues performed based on insulin signaling pathway proteins, and Cer 16:0, Cer 18:0, Cer 24:0, and sphingolipids subclasses’ abundance. Green and pink points indicate insulin signaling proteins and sphingolipid subclasses, respectively.

The associations between the insulin signaling pathway and the sphingolipid concentrations, as assessed by the loading plots of the PCA showed that most of the insulin signaling proteins were positively associated with Cer 16:0, Cer:SM ratio, total dhCer, and Cer-P, and negatively associated with Cer 18:0, total Spho, total Spha, and total GlcCer in the liver (Figure 4A). In the muscle, most of the insulin signaling proteins were positively associated with total SM, total dhCer-P, Cer16:0, and Cer 18:0, while the p-PKB ratio and mTOR showed a negative association with these compounds (Figure 4B). In the adipose tissue, while the p-mTOR ratio and AMPK showed positive associations with each other, InsR, mTOR, and p-AMPK ratio showed negative associations with several sphingolipid subclasses including total SM, total GlcCer, total LacCer, total Spho, and total Spha (Figure 4C).

## Discussion

These findings contribute to our understanding of the pathogenesis of metabolic dysregulation, including an unresolved metabolic inflammation, associated with the chronic surplus of dietary energy and protein intake. Although the experiment was done in fattening bulls, our findings may also be relevant for other species, including humans, regarding the proposed molecular level associations of insulin resistance and metabolic inflammation. Intensively fed obese bulls expressed a compensated disturbance of the insulin-glucose homeostasis reflected by euglycemic hyperinsulinemia. This systemic insulin insensitivity was based on reduced insulin signaling in insulin-sensitive tissues with tissue-specific patterns. Concomitantly, ceramide metabolism was stimulated by high dietary energy and protein intake resulting in intermediates, which are known to interact with the cellular insulin signaling cascade. As a major clinical outcome, this metabolic condition was associated with chronic inflammatory insults of the claw’s corium causing chronic intermittent phases of laminitis. In the following sections, interactions between the diet, insulin signaling, and ceramide metabolism are discussed. Further, underlying pathomechanisms of unresolved metabolic inflammation are proposed as a working hypothesis for future research about metabolic inflammation in humans and animals.

### Hepatic metabolic responses to high dietary energy and protein intake

Hepatic insulin signaling was interrupted at an early step of the signaling cascade; PKB phosphorylation was decreased by high dietary energy and protein intake, despite its higher total protein abundance. Consequently, downstream targets were less stimulated. However, the basal protein expression of PKB, mTOR, and AMPK was higher, most likely expressing a compensatory response to reduced PKB phosphorylation. Among the sphingolipids, Cer 16:0 and total dhCer-P had increased levels in the liver of HEP bulls. These metabolites are derived from *de novo* sphingolipid metabolism and their flux rate depends on free fatty acid availability (Holland and Summers, 2008). Dihydroceramide synthases produce dhCer from sphinganine (Pewzner-Jung et al., 2006), which can be phosphorylated to dhCer-P. Further, dhCer is a precursor for ceramide synthesis. Among the ceramides, Cer16:0 and Cer 18:0 are intermediates with proven detrimental effects on insulin signaling (Meikle and Summers, 2017). Ceramides are phosphorylated by ceramide kinase, and Cer-Ps are then involved in the eicosanoid synthesis (Lamour and Chalfant, 2005). Ceramides are known to decrease PKB phosphorylation by mainly targeting protein phosphatase 2A (PP2A) in different types of cells; however, the modulation of insulin signaling by ceramides is more complex than only through PP2A activation (Holland and Summers, 2008). A reduced PKB phosphorylation was also observed in the liver of fattening bulls, most likely due to higher hepatic ceramide concentrations, especially higher Cer 16:0 concentrations. Considering all bulls, the loading plot (Figure 4A) demonstrated a negative association between pPKB and Cer 16:0, but also with total dhCer, total dhCer-P, and the total Cer:SM ratio. These observations confirm the relationship between ceramide metabolism and insulin signaling in ruminants (McFadden and Rico, 2019). Thus, the hepatic metabolism of these bulls contributed to the systemic insulin insensitivity, presumably by causing an increased pancreatic insulin secretion or a reduced insulin turnover as a compensatory response. In humans, a higher rate of Cer production and Cer accumulation are associated with the development of insulin resistance and type 2 diabetes; however, the exact dysregulatory processes of lipid metabolism are not yet fully deciphered (Meikle and Summers, 2017).

The serine/threonine kinase mTOR is known to play a vital role in protein, glucose, and lipid metabolism (Mao and Zhang, 2018). Furthermore, mTOR function is strongly interrelated with AMPK function (Mao and Zhang, 2018). Both proteins, mTOR and AMPK were increased in their amount, however, their ratios of phosphorylated to total protein were equal in the LEP and HEP bulls. Thus, despite reduced PKB phosphorylation, the higher amount of total protein with maintained phosphorylation ratios suggested that these downstream targets were still activated at a higher level in HEP. Since mTOR stimulates glucose uptake and glycolysis by modulating the transcription factor hypoxia-inducible factor (HIF) 1 alpha (Düvel et al., 2010), that stimulation might contribute to maintaining euglycemia in HEP bulls. The lipogenic effect of mTOR activation leading to hepatosteatosis might be less biologically relevant in ruminants than in humans, since *de novo* lipogenesis is a feature of the adipose tissues in ruminants, and it is based on acetate utilization instead of glucose (Chilliard, 1993).

### Muscle and adipose metabolic responses to high dietary energy and protein intake

The expression of insulin receptor protein was not significantly affected in the muscle of HEP bulls; however, a significant decrease in insulin receptor expression was observed in the retroperitoneal adipose tissue of HEP bulls, compared with LEP. Associated changes in the sphingolipidome revealed that the HEP muscle had lower total SM, total dhCer, and total dhCer-P concentrations. While the association between sphingolipid metabolism and insulin resistance in skeletal muscle is well documented (Bandet et al., 2019), previous studies found that fatty acid-evoked myotubular ceramide accumulation and insulin insensitivity were rather correlated but independent events (Pillon et al., 2018). Accordingly, insulin signaling protein expression and their extent of phosphorylation were not significantly affected in the muscle of the HEP bulls, despite the differences in ceramide concentrations compared to LEP bulls.

The HEP bulls had higher Cer 24:0, total hexosylceramides, total sphinganines, and total sphingosines concentrations in their subcutaneous adipose tissue. Technically, insulin signaling protein expression was not measured in the subcutaneous but in the retroperitoneal adipose tissue due to the lack of sufficient sample material. However, we consider that the findings of both subcutaneous and retroperitoneal depots can be combined because the protein abundance of insulin signaling components, especially basal expression of the insulin receptor, were found to be equal in both depots in Holstein dairy cows (Kinoshita et al., 2016; Kenéz et al., 2019).

Thus, assuming an equal expression of the insulin receptor in these adipose depots in male Holstein cattle too, alterations in hexosylceramide levels appeared to affect insulin receptor expression. In 3T3-L1 fibroblast cultures, an increase in glucosylceramides inhibited insulin signaling by reduction of PKB phosphorylation after differentiation into mature adipocytes (Chavez et al., 2014). While PKB phosphorylation was not affected in the bulls herein, the insulin receptor expression was reduced in association with higher hexosylceramides. This effect was likely based on an interaction of hexosylceramides and the downstream GM3 gangliosides with the insulin receptor. Hexosylceramides were able to bind non-covalently to a lysine residue (Lys944) of the insulin receptor, just above the transmembrane domain, resulting in a disruption of the connection between membrane anchor proteins (adipocyte-specific caveolins) and the insulin receptor (Inokuchi, 2011). This was discussed as a membrane microdomain (lipid raft) disorder concept underlying metabolic disorders such as insulin resistance, resulting in a lower abundance of functioning insulin receptors in adipocytes (Inokuchi, 2011). Hexosylceramide metabolism was likely stimulated by enhanced inflammatory cytokine production, which was presumably associated with the chronic laminitis in the HEP bulls. In support of this conclusion, several inflammatory cytokines such as IL6 and TNF-α were increased in the plasma of cattle suffering from pasture-associated subclinical laminitis (Zhang et al., 2020). Higher sphinganine levels might reflect the higher availability of long-chain fatty acids for the *de novo* adipose ceramide metabolism, thereby stimulating ceramide synthesis and accumulation (Rico et al., 2016). In human subcutaneous adipose tissue, obese and diabetic individuals expressed lower sphinganine and total Cer concentrations, but sphingosines were not affected (Błachnio-Zabielska et al., 2012). These species-specific differences might be explained by metabolic differences between obese and insulin-resistant humans and bulls, respectively, which was also indicated by higher free fatty acid concentrations in the blood and enhanced dhCer concentrations in the adipose tissue of humans, but not in bulls. Thus, the potential role of lipotoxicity in the pathogenesis of diet-induced metabolic inflammation and insulin insensitivity, as stated for the human chronic over-eating syndrome (Engin and Engin, 2017), was not confirmed in bulls. Nevertheless, high Cer 24:0 concentration in plasma was associated with obesity and diabetes in humans (Jiang et al., 2013). Thus, in HEP bulls, high Cer 24:0 concentrations in adipose tissue may also signal the obese and insulin insensitive status.

### Unresolved metabolic inflammation in bulls in response to nutritional overload—a hypothesis

**FIGURE 5:**
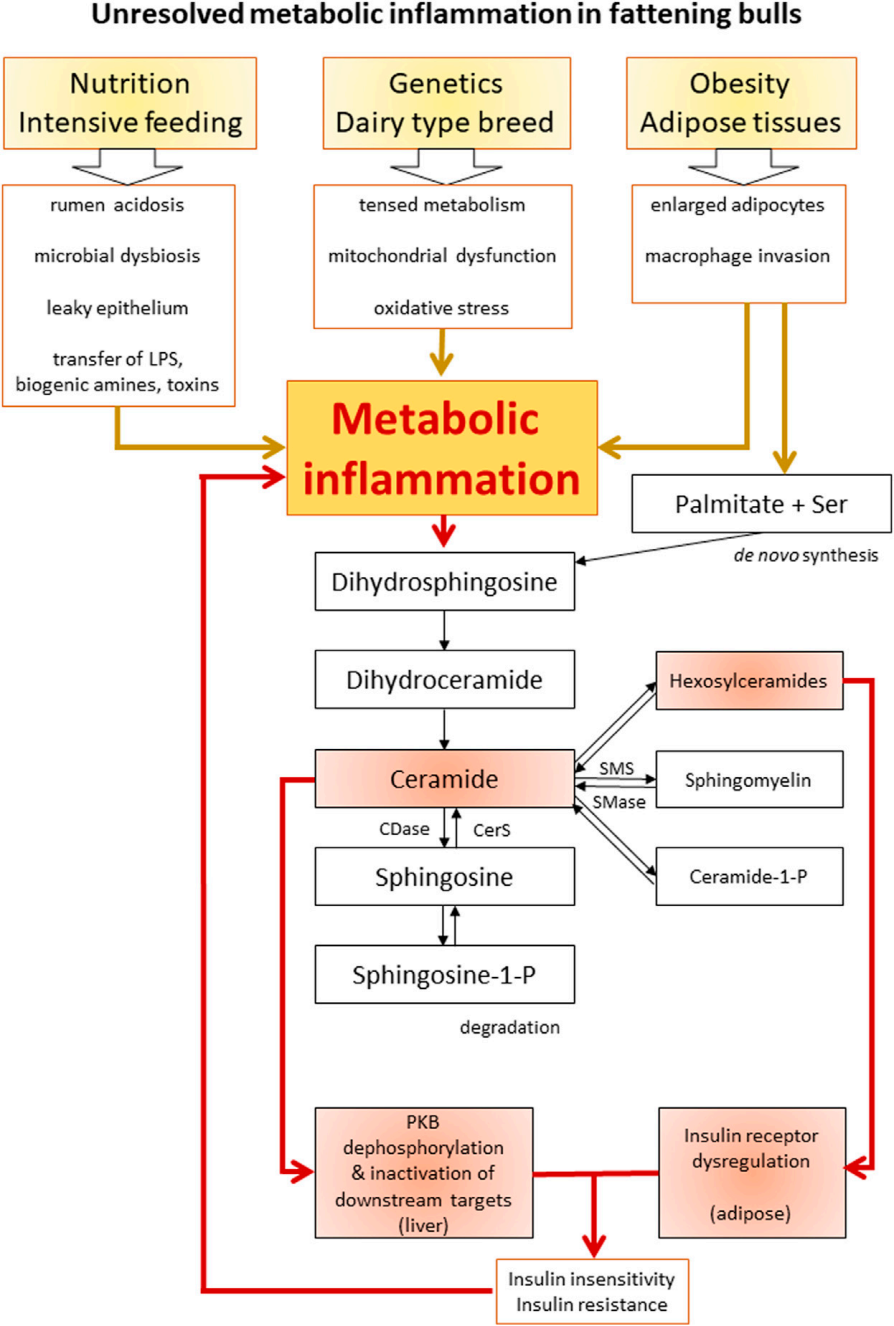
Schematic overview of the proposed interactions between inflammation, ceramide biosynthesis, and insulin resistance. Boxes highlighted in red indicate the scientific findings of this study. LPS, lipopolysaccharide; Ser, serine; CDase, ceramidase; CerS, ceramide synthase; SMS, sphingomyelin synthase; SMase, sphingomyelinase; PKB, protein kinase B.

Metabolic inflammation was characterized as a chronic low-grade inflammatory stage in dairy cattle (Bradford and Swartz, 2020). In the following section, a novel hypothesis about the pathogenesis of an unresolved metabolic inflammation in cattle is discussed based on the findings of the Holstein bulls herein and literature data (Figure 5).

Intensive feeding for high milk performance of dairy cows, as well as for enhanced growth and fattening of bulls, is one of three suggested origins of metabolic inflammation. Starch- and protein-rich diets create challenging conditions for ruminal fermentation. Subclinical rumen acidosis is a common disease in dairy and beef cattle, resulting in disturbed composition and metabolism of the residing microbiota (Owens et al., 1998; Petri et al., 2013; Fiore et al., 2020). As a consequence, the barrier function of rumen epithelium is affected, and biogenic amines, exo-, and endotoxins such as lipopolysaccharides (LPS) can enter the circulation, promoting systemic inflammation (Owens et al., 1998; Gozho et al., 2006). Particularly LPS causes inflammatory responses *via* Toll-like receptor 4 and activation of the nuclear factor kappa B pathway (Chaurasia et al., 2020), promoting metabolic inflammation. A second potential origin for metabolic inflammation is the genetic background of high-performing cattle. Holstein dairy cows are bred for high milk yield; thus, they are highly active in the synthesis and secretion of proteins, fats, and carbohydrates. Accordingly, male Holstein cattle are less genetically determined to gain protein (kg/d) for increasing body mass compared to beef breeds such as Angus and Angus × Simmental cross-breeds (Perry et al., 1991). Intensive feeding was not only a burden for a healthy rumen function, but also for the whole body metabolism due to an overload of nutrients. Since energy and amino acids could not be efficiently stored in the form of protein, fat accretion increased instead. Furthermore, the lack of physical activity reduced mitochondrial activity to produce ATP despite the high availability of nutrients, leading to inefficient mitochondrial function and generation of reactive oxygen species (ROS) in humans (Liepinsh et al., 2020). Oxidative stress due to intensive production promoted metabolic inflammation in farm animals (Lauridsen, 2019). This condition is similar to the setting in humans affected by a sedentary lifestyle and over-nutrition (Engin and Engin, 2017). The basic genetic design of modern-day humans is still considered to suit an ancient hunter and gatherer lifestyle, in which metabolism can easily cope with fasting but less potential is available to deal with a surplus of nutrients (Hotamisligil, 2006; Crittenden and Schnorr, 2017). As the third origin of metabolic inflammation, enlarged fat depots are known to secrete pro-inflammatory adipokines contributing to insulin resistance and metabolic inflammation (Sadri et al., 2010). While high non-esterified fatty acid (NEFA) load on the liver due to excessive adipose lipolysis was also responsible for metabolic inflammation in dairy cows, no increase in circulating NEFA was observed in the bulls suffering from chronic laminitis (Bäßler et al., 2021). However, even in the absence of high lipolytic activity, the secretory activity of large adipocytes that are filled with saturated fatty acids in intensively fed bull could still contribute to metabolic inflammation. So far, the secretory profile of pro-inflammatory signaling molecules that originate from hypertrophic adipose tissues of fattening bulls has not yet been characterized.

To summarize, a high amount of available saturated fatty acids, oxidative stress by inefficient mitochondria, and proinflammatory factors from rumen and adipocytes can be the main factors driving ceramide accumulation and metabolic inflammation. The enhanced synthesis of Cer decreases mitochondrial efficiency, blocks lipolysis, and reduces insulin signaling tissue-specifically. Further downstream in the sphingolipid pathways, hexosylceramides, SM, and Cer-P are also involved in metabolic disturbances (Summers et al., 2019). In the bulls, hexosylceramides were associated with decreased insulin receptor amount in adipose tissue, while Cer was linked with reduced PKB phosphorylation in the liver. The resulting systemic insulin insensitivity was compensated by hyperinsulinemia. Tissue level insulin resistance is known to be closely associated with metabolic inflammation expressing mitochondrial dysfunction, oxidative stress, and disturbed lipid metabolism (Hotamisligil, 2006); the latter two are factors that also promote metabolic inflammation. Thereby, ceramide synthesis was further enhanced in the bulls as a consequence of a positive but detrimental feedback mechanism. Adipose tissues may play an important role in connecting ceramide metabolism, insulin resistance, and inflammation in diet-induced obesity in humans (Chaurasia et al., 2020), as well as in intensively fed bulls exposed to a chronic nutrient surplus. Ceramides further promote insulin insensitivity and mitochondrial dysfunction, thereby strengthening the inflammatory state. This condition can represent a self-reinforcing cycle of unresolved metabolic inflammation in the bulls of this study (Figure 5). Under this condition of unresolved metabolic inflammation, homeostasis cannot be maintained, and the risk of metabolic diseases is increased (Bradford et al., 2015). In the intensively fed bulls, this risk manifested in chronic laminitis.

## Data Availability

The original contributions presented in the study are included in the article/Supplementary Material, further inquiries can be directed to the corresponding authors.
