# Supplementary material for: Ceramide metabolism associated with chronic dietary nutrient surplus and diminished insulin sensitivity in the liver, muscle, and adipose tissue of cattle
Source: Front Physiol. 2022 Aug 8;13:958837. doi: 10.3389/fphys.2022.958837 (PMC9393214; doi:10.3389/fphys.2022.958837)
Supplement: Supplementary file 1 [file DataSheet2.PDF]

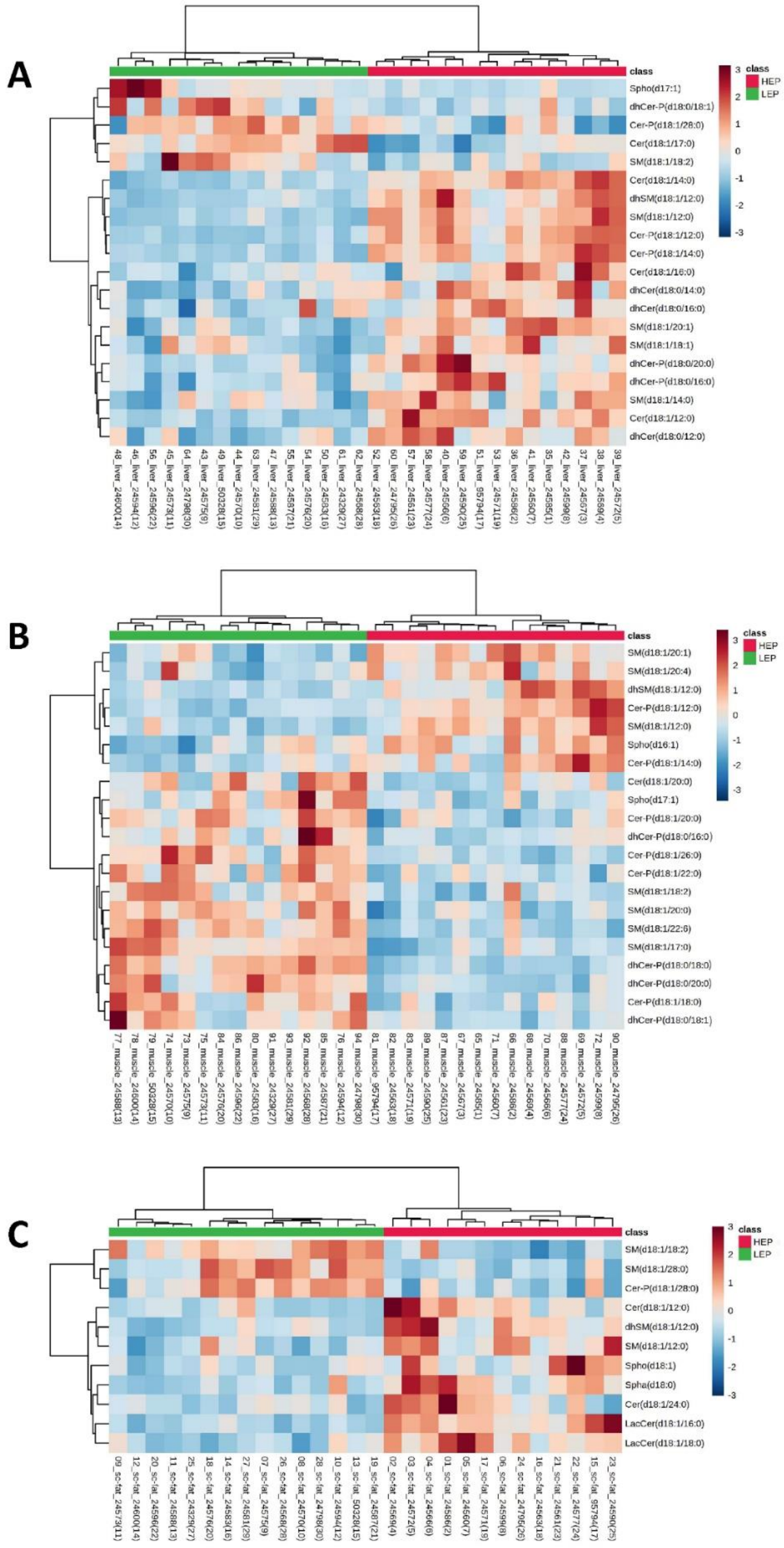

Supplementary Figure S2. Relative concentrations of sphingolipids that were significantly different between HEP and LEP in **(A)** liver (20 significant metabolites), **(B)** muscle (21 significant metabolites), and **(C)** subcutaneous adipose tissue (11 significant metabolites). Spha: sphinganines; dhCer: dihydroceramides; dhCer-P: dihydroceramide-1-phosphates; Cer: ceramides; Cer-P: ceramide-1-phosphates; LacCer: lactosyl-ceramides; SM: sphingomyelins; dhSM: dihydrosphingomyelins; Spho: sphingosines.
