# Supplementary material for: Ceramide metabolism associated with chronic dietary nutrient surplus and diminished insulin sensitivity in the liver, muscle, and adipose tissue of cattle
Source: Front Physiol. 2022 Aug 8;13:958837. doi: 10.3389/fphys.2022.958837 (PMC9393214; doi:10.3389/fphys.2022.958837)
Supplement: Supplementary file 2 [file DataSheet3.PDF]

Ceramide metabolism associated with chronic dietary nutrient surplus and diminished insulin sensitivity in the liver, muscle, and adipose tissue of cattle

Ákos Kenéz[1], Sonja Christiane Bäßler[2], Ezequiel Jorge-Smeding[1], Korinna Huber[2]

[1]Department of Infectious Diseases and Public Health, Jockey Club College of Veterinary Medicine and Life Sciences, City University of Hong Kong, Hong Kong SAR, China

[2]Institute of Animal Science, Faculty of Agricultural Sciences, University of Hohenheim, 70599 Stuttgart, Germany

Supplementary Table S1. Ingredients and chemical composition of total mixed rations (TMR) fed during the experimental period, as published previously (Bäßler et al., 2021)

	Diet	
	**HEP**	**LEP**
**Ingredients** (% of DM)		
Grass silage	40.33	69.99
Corn silage	13.68	29.92
Concentrate feed	45.99	0
Salt	0	0.09
**Chemical composition** (g/kg of DM unless noted otherwise)		
DM, g/kg	510	369
ME, MJ/kg of DM	11.4	10.2
Crude protein	155.0	110.0
Crude fat	40.0	37.0
Total sugar	31.0	20.0
Total starch	297.0	123.0
Crude fiber	171.0	239.0
Neutral detergent fiber	356.0	428.0
Acid detergent fiber	174.0	232.0
Ash	82.0	85.0
Ca	6.6	5.2
P	3.6	2.7
Na	1.4	1.0
K	18.8	24.1
Mg	2.2	2.1

HEP: high energy and protein diet; LEP: low energy and protein diet; DM: dry matter; ME: metabolizable energy
