# Supplementary material for: Ceramide metabolism associated with chronic dietary nutrient surplus and diminished insulin sensitivity in the liver, muscle, and adipose tissue of cattle
Source: Front Physiol. 2022 Aug 8;13:958837. doi: 10.3389/fphys.2022.958837 (PMC9393214; doi:10.3389/fphys.2022.958837)
Supplement: Supplementary file 3 [file DataSheet1.PDF]

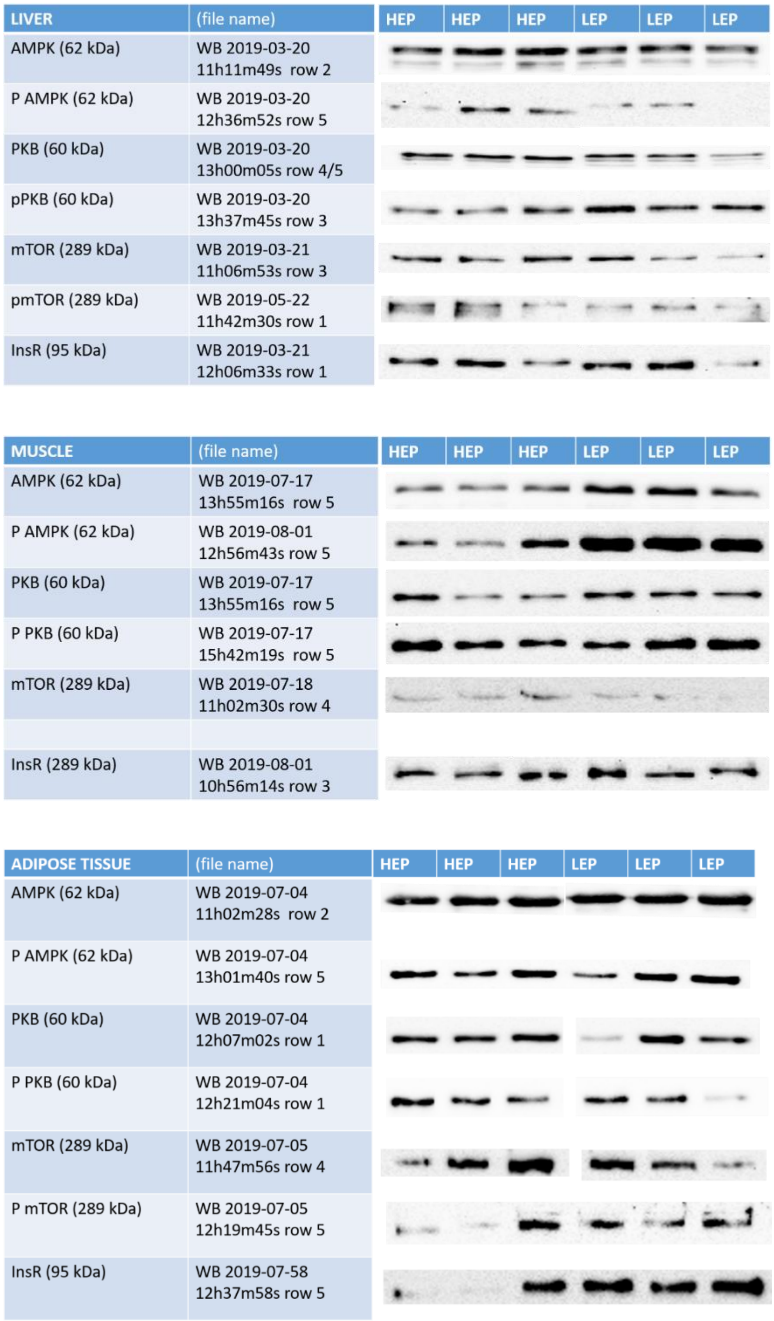

Supplementary Figure S1. Representative Western blot membrane images of the liver, muscle, and retroperitoneal adipose tissue of bulls fed a high energy and protein (HEP) or a low energy and protein (LEP) diet. Western blotting targeted the expression and phosphorylation of insulin signaling proteins: insulin receptor β (InsR), mechanistic target of rapamycin (mTOR), phosphorylated mTOR (P mTOR), protein kinase B (PKB), phosphorylated PKB (P PKB), 5’ adenosine monophosphate-activated protein kinase α (AMPK), and phosphorylated AMPK (P AMPK).
